# Maternal Aflatoxin Exposure, Birth Outcomes, and Infant Growth in Uganda

**DOI:** 10.4269/ajtmh.25-0426

**Published:** 2026-05-07

**Authors:** Lynne M. Ausman, Grace Namirembe, Julieta Mezzano, Jacqueline M. Lauer, Robin Shrestha, Edgar Agaba, Bernard Bashaasha, Jeffrey K. Griffiths, Elizabeth Marino-Costello, Jia-Sheng Wang, Juergen G. Erhardt, Andrew T. Gewirtz, Christopher P. Duggan, Patrick Webb, Shibani Ghosh

**Affiliations:** ^1^ Friedman School of Nutrition Science and Policy, Tufts University, Boston, Massachusetts, USA;; ^2^ Department of Public Health and Community Medicine, Tufts University, School of Medicine, Boston, Massachusetts, USA;; ^3^ Sargent College of Health & Rehabilitation Sciences, Boston University, Boston, Massachusetts, USA;; ^4^ Stellenbosch University, South Africa;; ^5^ Department of Agribusiness and Natural Resource Economics, Makerere University, Kampala, Uganda;; ^6^ Department of Environmental Health Science, University of Georgia, Athens, Georgia, USA;; ^7^ University of Hohenheim, Institute of Biological Chemistry and Nutrition, Stuttgart, Germany;; ^8^ Institute for Biomedical Sciences, Georgia State University, Atlanta, Georgia, USA;; ^9^ Division of Gastroenterology, Hepatology and Nutrition, Boston Children’s Hospital, Boston, Massachusetts, USA;; ^10^ Division of Nutritional Sciences, College of Human Ecology, Cornell University, Ithaca, New York, USA

## Abstract

The association between maternal aflatoxin exposure and infant anthropometric birth and growth outcomes was investigated in the present study, controlling for possible confounders*.* Pregnant women (*N* = 1,210) from 16 Ugandan subcounties were enrolled in a birth cohort study to track birth outcomes and subsequent growth of infants. Serum concentrations of aflatoxin B_1_ (AFB_1_)-lysine adduct, environmental enteric dysfunction markers of anti-lipopolysaccharide and anti-flagellin IgG and IgA, and markers of systemic inflammation, alpha-1 acid glycoprotein, and C-reactive protein were measured in mothers at birth and infants at 6 months of age. A generalized estimating equations model with an exchangeable correlation matrix was used to assess associations between maternal AFB_1_ blood concentration and weight, length, weight-for-age (WAZ), length-for-age (LAZ), and weight-for-length (WLZ) Z scores. Multivariable linear and logistic regressions were used to assess the association between infant aflatoxin concentrations and growth outcomes at 3 to 6 months of age. Serum aflatoxin concentrations in women at parturition were associated with reduced birth weight (*P* = 0.037) and WAZ (*P* = 0.034), but not with other birth outcomes. Aflatoxin concentrations in infants 6 months of age were not associated with changes in weight, height, WAZ, LAZ, or WLZ between 3 and 6 months of age. The present study confirmed an association between maternal aflatoxin and specific birth outcomes, but not between infant serum aflatoxin and infant early growth, which may be due to low exposure to aflatoxin-contaminated foods in early life. This finding highlights the importance of promoting national policy actions that minimize aflatoxin contamination of local food supplies, both on farms and in markets.

## INTRODUCTION

Mean stunting and wasting prevalence rates for children under 5 years of age in Uganda in 2022 were 23.4% and 3.6%, respectively.^1^ Despite concerted global efforts to uncover the specific causes of each form of undernutrition, a comprehensive understanding of the various factors remains elusive. The ubiquity of mycotoxins, which are naturally occurring toxic compounds produced by fungi on crops, has been widely acknowledged as a possible contributing factor.

The toxicity associated with mycotoxins began appearing in the scientific literature in the 1960s, with the identification of turkey-X-disease in England in 1961, a condition ultimately linked to groundnut meal contaminated with a type of mycotoxin termed “aflatoxin.”^2^ To date, more than 450 different types of mycotoxins and their metabolites have been identified as causes of acute or chronic diseases in animals and humans.^3^ Investigation into the various types of mycotoxins, the crops that they infect, and the conditions conducive for fungal growth is an ongoing effort involving multiple stakeholders, including farmers, animal husbandry personnel, and the health care system.^4^^–^^8^

Aflatoxin B_1_ (AFB_1_)-lysine adduct is one of the best described of these toxins; the crops it infects and the toxin metabolites produced in mammalian systems have been well documented ^9^^,^^10^ The genotoxic effects of aflatoxins are due to a metabolite of AFB_1_, namely 8,9-exo-epoxide and 8,9-endo-epoxides formed in the CYP450 superfamily, primarily in the liver. These bind to DNA, RNA, and proteins, forming adducts. The result is inhibited RNA, DNA, and protein synthesis in all major organs of the body.^9^ Aflatoxin in high quantities is associated with various cancers, as well as death.^11^ Even in small amounts, it has been associated with carcinogenic, mutagenic, and genotoxic effects on major body organs.^9^

There is some evidence that aflatoxin could be associated with poor birth and growth outcomes of infants.^12^^–^^15^ In a narrative review, Gorain and colleagues^16^ have recently summarized numerous studies on aflatoxin exposure during pregnancy and infancy and their association with health outcomes. Research suggests that mycotoxins compromise intestinal barrier function through a variety of toxic effects on intestinal cells. This can lead to nutrient malabsorption, increased intestinal permeability, intestinal and systemic inflammation, and potentially poor growth outcomes in infants and young children.^17^^,^^18^ Thus, it is hypothesized that the growth-inhibiting properties of aflatoxin would be reflective of stunting, underweight, and wasting in infants and young children. Several studies have addressed this hypothesis, but results are mixed.^19^^–^^21^

The two specific aims for the current study were to 1) determine if maternal aflatoxin concentrations during pregnancy, adjusted for inflammation markers and other maternal characteristics, were associated with impaired infant birth outcomes (birth weight, length, weight-for-age [WAZ], length-for-age [LAZ], weight-for-length [WLZ], small-for-gestational age [SGA] status, and preterm status) in a cross-sectional analysis, and 2) determine if infant aflatoxin concentrations, adjusted for environmental enteric dysfunction (EED) biomarkers and other maternal characteristics, were associated with growth impairment (g/day) or daily changes in anthropometrics between 3 and 6 months of age in a longitudinal analysis.

## MATERIALS AND METHODS

### Study design.

The Uganda Birth Cohort Study was a prospective birth cohort study conducted between 2014 and 2016 by the Feed the Future Innovation Laboratory for Nutrition, hosted by Tufts University, in which more than 5,000 pregnant women and their infants were followed up for several months after birth.^22^^–^^25^ The current analysis is based on a substudy sample (*N* = 1,210) of the full cohort of mother–infant dyads. Mothers were sampled over a period of several months, with 54.7% of births occurring during the wet period. Data on 1,210 mothers were available, including serum aflatoxin levels, inflammatory biomarkers, maternal age, education, diet, and household characteristics. Birth outcome data and anthropometry data at birth on their infants were also available (*N* = 1,210). In addition, from the original subsample of 1,210, 473 infant samples collected at 6 months of age were analyzed for serum aflatoxin levels, inflammatory biomarkers (α-1 acid glycoprotein [AGP] and C-reactive protein [CRP]), and EED biomarkers (anti-lipopolysaccharide [LPS] and anti-flagellin IgG and IgA). The growth rate (rate of change in body weight) and the rates of change in the anthropometric indices of those subsampled infants between 3 and 6 months of age were also examined. A detailed consort diagram of the overall study sample has been presented previously.^25^

Study participants lived in 16 subcounties in rural northern and southwestern Uganda. Trained enumerators conducted in-person interviews, gathering information through questionnaires from mother–infant dyads during pregnancy, at childbirth, and at 3 and 6 months of age. Data covered various aspects, including economic status, health, disease, diet, agricultural practices, incomes and expenditures, and educational variables within the family unit. Additionally, anthropometric measurements were taken at each time point, and blood samples for specific biochemical markers were collected from the mother at birth and from the infants at 6 months of age.^23^

### Measurements.

Maternal height and infant length were measured at parturition to the nearest 0.1 cm using a portable height board (ShorrBoard infant/child/adult portable height-length measuring board; Weight and Measure, LLC, Olney, MD). Weight was measured to the nearest 0.1 kg using an electronic scale (Seca model 874; Seca Corporation, Hamburg, Germany). Maternal mid-upper arm circumference (MUAC; cm) was obtained using a measuring tape at the point of delivery. Anthropometric measurements were taken in triplicate for each participant, and the averaged values were used for analysis.

Hemoglobin concentrations were measured using a finger-prick blood sample with a portable hemoglobinometer (HemoCue 301; HemoCue America, Brea, CA). Values <11 g/dL were considered indicative of anemia.^26^ Trained phlebotomists collected blood via venipuncture into Becton Dickinson Vacutainer tubes (Becton, Dickinson and Company, Franklin Lakes, NJ). Samples were transported on ice to a laboratory facility in Kampala, where serum was separated, aliquots were prepared for various analyses, and the samples were frozen at –80°C. Samples were shipped from Kampala to European and U.S. destinations using an international carrier with dry ice and temperature control verification in each box.

The Household Food Insecurity Access Score (HFIAS; a continuous variable) and the Household Food Insecurity Access Prevalence (a categorical variable) were assessed according to the procedure outlined in Coates et al.^27^ The safety of the drinking water was measured with a compartment bag test using methods already described.^28^ Drinking water was considered contaminated if *Escherichia coli* was present at a concentration >1/100 mL of water.^29^

### Laboratory analysis.

Serum samples were analyzed to detect AFB_1_-lysine adducts.^30^ Aflatoxin detection was performed via high performance liquid chromatography using a fluorescence-validated method at the University of Georgia.^31^^,^^32^ The lower limit of detection (LOD) for this method is 0.4 pg AFB_1_-lysine per mg albumin, and the recommendation to impute half (0.2 pg AFB_1_-lysine/mg albumin) of the values that fell below the LOD was followed.^14^ Biomarkers of inflammation (CRP and AGP) were analyzed using a sandwich ELISA by the VitMin Laboratory in Willstaett, Germany.^33^ When AGP levels >1 g/L or CRP levels >5 mg/L were detected, samples were considered “inflamed.”^34^ Biomarkers of EED included anti-LPS and anti-flagellin IgG and IgA.^35^ These biomarkers are translocation markers, indicating response to the passage of bacteria from the lumen of the intestine, across the epithelial barrier, and into the systemic circulation; they were analyzed in serum samples at Georgia State University (Atlanta, GA) using previously described ELISA methods.^28^

## STATISTICAL ANALYSES

Length-for-age, WAZ, and WLZ were calculated using the WHO Multicenter Growth Reference Study growth standards.^36^ Outliers, defined as LAZ <−6 or >6, WAZ <−6 or >5, or WLZ <−5 or >5, were set to missing. Preterm birth was defined as <37 weeks’ gestation,^37^ and SGA was defined as infants who were small for gestational age and sex (weight centiles below the 10th percentile) using INTERGROWTH-21 standards.^38^ Because the flagellin and LPS markers yielded similar results, LPS IgG served as a proxy for all of them. Inflammation was defined as having AGP levels >1 g/L or CRP levels >5 mg/L.

Because of their skewed distribution, aflatoxin and EED biomarkers underwent a natural logarithmic transformation before statistical analyses were conducted. To evaluate the association between maternal aflatoxin concentrations and birth outcomes (birth weight, length, LAZ, WAZ, WLZ, SGA status, and preterm status) in 1,210 mother–infant dyads, a generalized estimating equations (GEE) model with an exchangeable correlation matrix was used (SAS, v 9.4; SAS Institute Inc., Cary, NC). Variables that were statistically significant at *P* <0.1 were considered potential confounders in bivariate associations. Confounders were also defined as variables of interest on the basis of literature reviews and a priori knowledge. Determinants included maternal age, maternal height, infant’s sex, household food security status (HFIAS), maternal anti-LPS IgG, inflammation (AGP levels >1 g/L or CRP levels >5 mg/L), maternal antenatal care visits, and subcounty clustering. For binary outcomes, GEE models with a logit link were used to investigate the association between birth outcomes and aflatoxin levels, whereas GEE models with an identity link were used for continuous outcomes, accounting for community clustering.

For the longitudinal studies, all 473 infants with body weight recorded at both 3 and 6 months of age and aflatoxin, AGP, and CRP concentrations, along with all covariates, recorded at 6 months of age were selected. All infants were confirmed to be exclusively breastfeeding at 3 months of age. The association between infant aflatoxin concentrations at 6 months of age and the change/day in anthropometric characteristics (LAZ, WAZ, and WLZ) and body weight between 3 and 6 months of age was examined. The growth models were adjusted for preterm status (<37 weeks’ gestation); maternal age, education, and height; food security; subcounty; infant sex; water quality (compartment bag test); and LPS IgG as a proxy for EED. Reduced sample sizes reflect the presence of one or more missing covariates.

Analyses were performed using SAS version 9.4 and STATA 14 software (StataCorp LLC, College Station, TX). For all analyses, a* P*-value <0.05 was considered statistically significant.

## RESULTS

Household, women, and infant characteristics are presented in Table 1. A total of 53% of households were from northern Uganda, and the vast majority were headed by males. Mothers were, on average, 27 years of age and had normal body composition, as indicated by the average MUAC measurement within the normal range. Slightly more than half (51%) had at least 6 years of formal education. Approximately 60% of households experienced some level of food insecurity, ranging from mild to severe. Approximately two-thirds lacked access to a clean water supply (not shown), and only 5.6% of the mothers tested positive for malaria. A small proportion of the women (13.4%) were anemic. Approximately half (48.5%) of the infants were male. Mean infant birth weight was 3.26 kg, and mean infant length was 47.6 cm; 26.3% of infants were stunted, 8.0% were wasted, and 2.4% were underweight at birth.

**Table 1:** Baseline household, women’s, and infants’ characteristics at childbirth in rural Uganda

Characteristic	*n*	Mean/%	SD	Min	Max
Household characteristics					
HFIAS^27^	1,190	–	–	–	–
Food secure	436	36.6	–	–	–
Mildly food insecure	317	26.6	–	–	–
Moderately food insecure	282	23.7	–	–	–
Severely food insecure	155	13.0	–	–	–
Region (north, %)	1,191	53.2	–	–	–
Household head sex (male, %)	1,191	94.4	–	–	–
Married/cohabiting (%)	1,191	95.3	–	–	–
Wealth index (mean)^39^	1,188	2.93	1.39	1.0	5.0
Season of AFB_1_ measurement (wet, %)*	1,210	54.7	–	–	–
Household size (>4 members, %)	1,182	65.0	–	–	–
Women’s characteristics					
Pregnancy term (full term, %)	1,080	80.1	–	–	–
Age (years)	1,181	26.9	6.2	16.0	47.0
Height (cm)	1,208	159.2	6.0	142.5	177.6
Education (years)	1,191	–	–	–	–
<6	583	49.0	–	–	–
6–11	585	49.1	–	–	–
≥12	23	1.93	–	–	–
MUAC (cm)	1,207	26.2	2.5	20.0	36.7
MDD-W (met, %)^†^	1,191	17.3	–	–	–
Malaria positive (%)	1,154	5.6	–	–	–
Anemia % (Hb <11 g/dL)	1,153	13.4	–	–	–
Infant’s characteristics					
Sex (male, %)	1,207	48.5	–	–	–
Length/height (cm)	1,210	47.57	3.33	36.83	60.0
Weight (kg)	1,210	3.26	0.49	1.50	6.07
WLZ^‡^	979	0.55	1.76	−4.86	5.00
WAZ^‡^	1,206	−0.12	1.01	−4.47	4.91
LAZ^‡^	1,201	−1.01	1.73	−5.90	5.34
Stunted, % (LAZ <−2)	1,207	26.3	–	–	–
Wasted, % (WLZ <−2)	992	8.0	–	–	–
Underweight, % (WAZ <−2)	1,206	2.4	–	–	–

AFB_1_ = aflatoxin B_1_; Hb = hemoglobin; HIFIAS = Household Food Insecurity Access Score; LAZ = length-for-age; MDD-W = Minimum Dietary Diversity-Women; MUAC = mid-upper arm circumference; WAZ = weight-for-age; WLZ = weight-for-length.

The variability in sample size is attributable to unknown maternal birth date, missing self-reported information needed for the Wealth Index, missing infant birth weight, or variables flagged and dropped during cleaning using the anthropometric software.

* Season of sample collection (the wet season occurs from March to May and from September to November).

^†^ Consumption of ≥5 food categories meets the MDD-W.^40^

^‡^ Length-for-age, WAZ, and WLZ were calculated using the WHO Multicenter Growth Reference Study growth standards.^36^

Aflatoxin, EED, inflammation, and anemia biomarkers for women at childbirth are presented in Table 2. These concentrations are a proxy for exposure of the infant in utero. The levels of maternal AFB_1_-lysine adduct varied widely from 0.2 pg/mg to 492.80 pg/mg serum albumin. The geometric mean was 4.35 pg/mg serum albumin. A total of 94% of the aflatoxin values fell on the standard curve. Approximately 50% of the mothers had AGP or CRP levels defined as “inflamed.” Additionally, biomarkers for EED, measured using anti-flagellin IgA and IgG and anti-LPS IgA and IgG, indicate significant environmental enteropathy, although no cutoffs have been assigned for these values.

**Table 2:** Biomarkers of women at childbirth in rural Uganda

	Women (At Childbirth)					
Biomarker	*N*	Mean/%	SD	CI	Min	Max
AFB_1_-lysine (pg/mg serum albumin)*	1,210	4.35	–	4.02 to 4.70	0.2	492.80
AFB_1_-lysine maternal detection rate (% detectable)	1,210	94.0	–	–	–	–
Inflamed (%)^†^	1,210	50.0	–	–	–	–
Anti-flagellin IgA^‡^	1,207	0.70	0.52	–	−3.04	2.62
Anti-flagellin IgG^‡^	1,208	0.83	0.36	–	−5.40	1.73
Anti-LPS IgG^‡^	1,206	0.80	0.36	–	−7.27	1.61
Anti-LPS IgA^‡^	1,205	0.69	0.45	–	−2.28	1.83
Anemia, % (Hb <11 g/dL)	1,153	13.4%	–	–	–	–

AFB_1_ = aflatoxin B_1_; Hb = hemoglobin; Ln = natural logrhythm; LPS = lipopolysaccharide.

* Geometric mean and 95% CIs reported for AFB_1_-lysine.

^†^ Mothers with α-1 acid glycoprotein levels >1 g/L or C-reactive protein levels >5 mg/L.^34^

^‡^ Ln-transformed values.

The relationships between maternal aflatoxin at childbirth and infant birth outcomes are presented in Table 3. Using the adjusted model, both birth weight (β: –0.0238; 95% CI: –0.0462 to –0.0014; *P* = 0.037) and WAZ (β: –0.0501; CI: –0.0963 to –0.0039; *P* = 0.034) were negatively associated with maternal aflatoxin levels at parturition. Maternal aflatoxin concentrations were not associated with LAZ, WLZ, SGA status, preterm birth, or stunting.

**Table 3:** Associations between maternal aflatoxin B_1_-lysine adduct levels and birth outcomes

	Unadjusted Model			Adjusted Model*		
Dependent Variables	*n*	β Estimate/OR	*P*-Value/95% CI	*n*	β Estimate/OR	*P*-Value/95% CI
Birth weight (kg)	1,210	−0.018	0.0692	1,159	−0.024	0.0374^†^
Birth length (cm)	1,210	−0.075	0.2744	1,159	−0.0713	0.1618
WAZ*	1,206	−0.045	0.0290^†^	1,158	−0.0501	0.0337^†^
LAZ*	1,201	−0.042	0.2361	1,154	−0.0390	0.1215
WLZ*	979	−0.066	0.0967	938	−0.0612	0.1333
Small for gestational age	979	1.109	0.970 to 1.266	959	1.108	0.945 to 1.298
Preterm	1,080	0.990	0.890 to 1.102	1,054	1.012	0.908 to 1.128
Stunting	1,207	1.078	0.984 to 1.182	1,159	1.056	0.986 to 1.130

LAZ = length-for-age; Ln = natural logrhythm; LPS = lipopolysaccharide; OR = odds ratio; WAZ = weight-for-age; WLZ = weight-for-length.

Each row represents the outcome used in a separate regression model. Aflatoxin B_1_-lysine levels were ln-transformed and reported as pg/mg albumin.

* Generalized estimating equation models were used for continuous outcomes with an identity link and for binary outcomes with a logit link, accounting for correlation within clusters. All of the following variables were adjusted for: subcounty clustering, maternal age, maternal height, inflammation (α-1 acid glycoprotein levels >1 g/L or C-reactive protein levels >5 mg/L), Household Food Insecurity and Access Scale, infant sex, antenatal care visits, and maternal anti-LPS IgG.

^†^ *P*-value is significant at <0.05.

To determine the association between aflatoxin concentration in children and growth rate (g/day) or changes in anthropometric indices, rather than a static time point, it was important to have longitudinal data on the anthropometrics of the infants and accurate information on their exposure during that period. Biomarkers were available for 473 infants at 6 months of age. These infants were determined to have been exclusively breastfed through at least 3 months of age. The AFB_1_ concentrations of the infants at 6 months of age are presented in Table 4. Although the range of the AFB_1_ concentrations was substantial, the geometric mean of 1.1 pg/mg serum albumin was approximately one-quarter that of the maternal concentration at birth.

**Table 4:** Biomarkers of infants 6 months of age in longitudinal study conducted in rural Uganda

	Infants (6 Months)					
Biomarker	*n*	Mean/%	SD	CI	Min	Max
AFB_1_-lysine (pg/mg serum albumin)*	473	1.11	−	0.99–1.25	0.2	378.13
Inflamed (%)^†^	473	49.68	−	−	−	−
Anti-flagellin IgA^‡^	472	−0.09	0.57	−	−2.31	2.47
Anti-flagellin IgG^‡^	473	0.61	0.25	−	−0.31	1.50
Anti-LPS IgG^‡^	473	−0.04	0.58	−	−3.61	1.74
Anti-LPS IgA^‡^	473	0.60	0.28	−	−0.41	1.56
Anemia, % (Hb <11 g/dL)	473	58.14	−	−	−	−

AFB_1_ = aflatoxin B_1_; Hb = hemoglobin; Ln = natural logrhythm; LPS = lipopolysaccharide.

* Geometric mean and 95% CIs reported for AFB1-lysine.

^†^ Infants with α-1 acid glycoprotein levels >1 g/L or C-reactive protein levels >5 mg/L.^34^

^‡^ Ln-transformed values.

**Figure 1.**
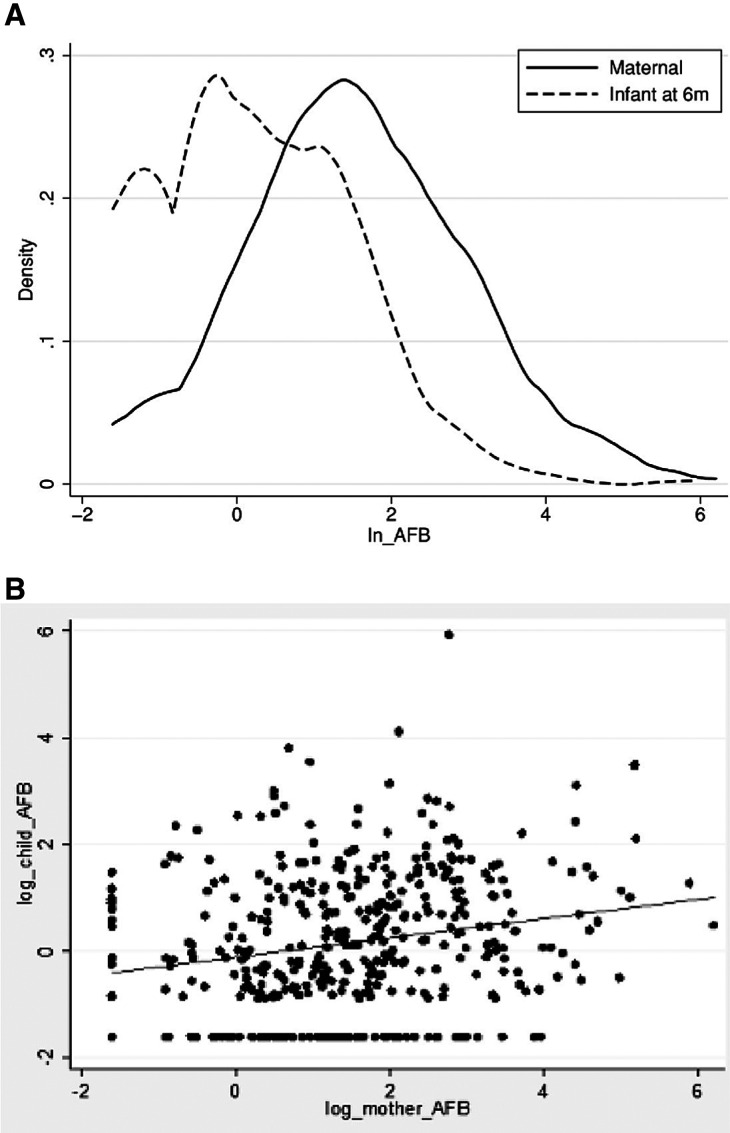
(**A**) Ln aflatoxin B_1_ (AFB_1_)-lysine adduct concentrations in 421 mother–infant dyads. (**B**) Association of maternal aflatoxin at birth and infant aflatoxin at 6 months of age (*P* <0.001; r = 0.2041). Of the 473 mother–infant dyads, AFB_1_-lysine adduct concentrations were available for 421.

A density plot of AFB_1_-lysine concentrations for mothers at childbirth and infants at 6 months of age for 421 available mother–infant dyads is shown in Figure 1A. The scatter plot for AFB_1_ ln-transformed values for these pairs (r = 0.2041; *P* = 0.0001) is shown in Figure 1B. Infant values were highly correlated with maternal levels.

Anthropometric data for the longitudinal study are presented in Table 5. There were 473 infants with biomarker values at 6 months of age who were exclusively breastfed until at least 3 months of age. To determine the aflatoxin exposure of these infants between 3 and 6 months of age, the following assumptions were made: 1) AFB_1_-lysine adduct on albumin has a half-life of ∼21 days^42^; 2) at 3 months (4.5 half-lives), only 5% of umbilical contribution would remain in the infant; and 3) the AFB_1_ concentration in breast milk would be constant over the period of 3 to 6 months. Therefore, the 6-month aflatoxin value would represent exposure over the 3- to 6-month period.

**Table 5:** Anthropometrics characteristics of infants between 3 and 6 months of age in a longitudinal study conducted in rural Uganda

	3 Months (*n* = 473)					6 Months (*n* = 473)				
Infants’ Characteristics*	*n*	Mean/%	SD	Min	Max	*n*	Mean/%	SD	Min	Max
Infants (male, %)	473	49.3	−	−	−	473	49.3	−	−	−
Length/height (cm)	421	58.3	3.53	45.56	66.10	473	63.82	3.57	51.37	77.00
Weight (kg)	421	5.87	0.86	2.2	9.70	473	7.24	1.02	4.10	10.85
WLZ	377	0.61	1.58	−4.22	4.91	463	0.52	1.48	−3.68	4.38
WAZ	395	−0.80	1.19	−5.31	2.95	467	−0.58	1.19	−5.09	2.51
LAZ	390	−1.61	1.66	−6	3	470	−1.37	1.56	−5.59	4.37
Stunted (%)^†^	390	35.9	−	−	−	470	34.2	−	−	−
Wasted (%)^†^	377	5.3	−	−	−	463	5.0	−	−	−
Underweight (%)^†^	395	13.7	−	−	−	463	10.1	−	−	−

LAZ = length-for-age; WAZ = weight-for-age; WLZ = weight-for-length.

* All infants were documented to have been exclusively breastfed from birth to 3 months of age.

^†^ Prevalence of stunting is defined as LAZ <−2, wasting is defined as WLZ <−2, and underweight is defined as WAZ <−2.^41^

To determine the association between aflatoxin concentration and growth, daily changes in weight, length, WAZ, LAZ, and WLZ over the 3- to 6-month period were calculated. The results are shown in Table 6. Whether unadjusted or adjusted (see footnotes), there was no observable association between infant serum AFB_1_-lysine adduct values and the variables assessed for infant growth.

**Table 6:** Infants’ aflatoxin B_1_-lysine adduct versus change in anthropometric indices between 3 and 6 months of age

	Unadjusted Model			Adjusted Model*		
Change/Day from 3 to 6 Months	*n*	β Estimate	*P*-Value	*n*	β Estimate	*P*-Value
Weight (kg)	371	0.000193	0.551	374	−0.0000921	0.791
Length (cm)	371	−0.000212	0.896	374	−0.121000	0.476
Weight-for-age Z score	366	−0.000313	0.476	368	−0.000023	0.962
Length-for-age Z score	363	−0.000146	0.846	366	0.000776	0.303
Weight-for-length Z score	349	−0.000586	0.452	351	−0.000428	0.590

Ln = natural logrhythm; LPS = lipopolysaccharide.

The sample size for these analyses varied because of missing data in the covariates.

* Adjusted for preterm status; maternal age, education, and height; food security; subcounty; infant sex; water quality^29^; and ln LPS IgG.

## DISCUSSION

The findings of the present study reveal an association between AFB_1_-lysine exposure during pregnancy and a decrease in both birth weight and WAZ score in Ugandan infants. The study was conducted over a 2-year period, with mothers entering the study at different times. Exposure to aflatoxin was presumed to have occurred during the mothers’ entire pregnancies. In this Ugandan population, the maternal median and geometric mean AFB_1_-lysine adducts were 3.12 pg/mg albumin and 4.35 pg/mg albumin, respectively. The detection rate of 94% at an albumin LOD of 0.4 pg/mg is comparable to that observed in most regions where maize and groundnuts are consumed.^14^^,^^43^ Similar findings that reveal a median AFB_1_-lysine adduct of 5.83 pg/mg albumin in Ugandan mothers mid-gestation were reported in a separate central region of Uganda (Mukono District).^15^ This effect on birthweight is in accordance with birth outcomes of pregnant women in Ghana,^44^ the Gambia,^45^ and the United Arab Emirates,^46^ where maternal aflatoxin concentrations were negatively associated with infant birth weight, as well as in Nepal in terms of SGA status.^14^

The present study’s findings, however, do not reveal an association between maternal aflatoxin AFB_1_-lysine adduct concentration and stunting (LAZ) or wasting (WLZ) of the infant at birth. In a cohort of Tanzanian children, Chen and colleagues^47^ demonstrated that the presence of mycotoxins was not associated with environmental enteropathy, indicating that the two may have distinct effects. There is an increasing number of studies conducted on maternal and infant EED and their associations with poor birth and growth outcomes.^29^^,^^48^^–^^50^ At a minimum, intestinal permeability can lead to malabsorption of important nutrients. Adjusting for the presence of EED in the current study may have helped decrease any effects attributed solely to aflatoxin.

Other important dietary, biological, and environmental characteristics may also play crucial roles. Mezzano et al.^25^ have shown significant negative associations between LAZ and vitamin A status (using retinol binding protein as a proxy) and between preterm birth and iron status (using serum serum transferrin receptor). Additionally, over the last few decades, with the national and international focus on the Sustainable Development Goals,^51^ Uganda has exhibited a steady improvement in health outcomes, with the prevalence of stunting declining from 44% in 1995 to 33% in 2011^52^ and 26.3% in the present cohort.

Given that the biological half-life of plasma AFB_1_-lysine bound to albumin is ∼3 weeks, maternal aflatoxin levels measured at birth are indicative of recent exposure (during the last trimester). Because fetal development begins in the earliest stages of pregnancy,^53^ and earlier maternal exposure is unknown, it is difficult to determine if there was sufficient time for any measurable effects to impact the growing fetus. This may explain the insignificant findings for some of the birth outcomes.

Much of the published literature on the effect of aflatoxin on children’s growth consists of cross-sectional studies conducted at a variety of time points throughout infancy and early childhood. In a systematic review of papers published between 2002 and 2018 on dietary exposure to mycotoxins and child growth,^19^ investigators found mixed results, leading to no firm conclusions. There is considerable heterogeneity across studies, including differences in measurement methods and indicators, exposure periods, seasonal variation, confounding factors, study populations, and sample sizes. More recently published data from longitudinal, rather than cross-sectional, studies reveal a significant negative association between aflatoxin concentration and changes in LAZ, length, knee–heel length, and WAZ over a span of almost 2 years.^54^ In addition, serum aflatoxin concentrations were associated with stunting.

It is important to differentiate studies conducted to investigate in utero effects, those conducted to assess infants solely during breastfeeding in the first few months, and those conducted to assess effects between 6 months and 5 years of age, a period marked by the introduction of complementary and family foods that increase the risk of AFB_1_ exposure.^45^^,^^55^^,^^56^ To study the effect of aflatoxin on growth, the effect of infant aflatoxin exposure on daily changes in body weight, length, and the three WHO indices (WAZ, WLZ, and LAZ) between 3 and 6 months of age, when infants were being breastfed, was assessed. Analyses were adjusted for preterm status; maternal age, education, and height; food security; subcounty; infant sex; water quality; and ln LPS IgG. No association was observed between aflatoxin and any of the change indices at 3 to 6 months of age. The degree of stunting, wasting, and underweight, as well as the three anthropometric indices, were similar at both 3 and 6 months of age. The sufficiently low or uniform infant geometric mean serum AFB1- lysine adduct levels (1.11 pg/mg albumin) observed in these breast-fed infants might explain a lack of measurable effect during this 3 month period. In addition, the high correlation between maternal aflatoxin levels at birth and infant values at 6 months (Figure 1) may indicate that complementary feeding was not a major contributing source of aflatoxin in these infants. In a cohort of Bangladeshi infants followed over 36 months, Mahfuz et al.^57^ showed that AFB_1_-lysine adduct values at 7 months of age in children who were largely breastfeeding were 0.71 pg/mg albumin (geometric mean), compared with 1.11 pg/mg (geometric mean) for the Ugandan infants in the current study at 6 months of age. In a longitudinal study in Banke, Nepal, aflatoxin concentrations and anthropometric biomarkers were assessed in children aged 3 to 22 months.^54^ Geometric mean AFB_1_-lysine levels were 0.86 at 6 months of age, similar to the AFB_1_ levels in the Ugandan infants in the present study. However, concentrations gradually increased over the following 14 to 18 months. These increases were associated with changes in length, LAZ, WAZ, and stunting, thereby reinforcing the importance of preventing contamination from complementary foods. In another longitudinal study conducted in children in Bhaktapur, Nepal, mean AFB_1_-lysine biomarkers averaged 3.62 pg/mg albumin over a period of 15 to 36 months of age, again providing evidence of chronic exposure to environmental aflatoxin.^58^ It could be that if the longitudinal portion of the current study had been conducted at later ages, when children would be consuming contaminated foodstuffs, an effect similar to that in the study from Nepal could have been observed.^54^

The present Ugandan birth cohort study is one of just a few in which the authors have attempted to determine the effect of aflatoxin while accounting for EED and systemic inflammation, which are associated with impaired growth and reduced height. Indeed, while controlling for EED, the presence of aflatoxin is still significantly associated with reduced birth weight and negatively associated with WAZ. In addition, the low levels of aflatoxin and the apparent lack of effect on infant growth between 3 and 6 months of age reinforce the importance of exclusive breastfeeding through at least 6 months of age.

### Limitations and strengths.

In the present study, the longitudinal design enabled an examination of children’s birth outcomes and growth indices over time relative to their aflatoxin exposure. Potential confounding variables were rigorously controlled for, including maternal, household, and environmental factors, enhancing the internal validity of the findings. The incorporation of diverse biomarkers, including anti-LPS and anti-flagellin IgG and IgA, systemic inflammation markers (CRP and AGP), and AFB_1_-lysine adduct concentrations, added depth to the assessment of EED and aflatoxin exposure. This comprehensive approach addresses some knowledge gaps regarding aflatoxin exposure and infant outcomes and growth. In summary, the present study contributes to a more thorough understanding of the relationship between aflatoxin exposure and infant birth outcomes and growth.

However, the study findings are subject to certain limitations. Data were missing for some variables, precluding their use in certain analyses. However, this should not have detracted from the overall interpretation of the analyses. In addition, the accuracy of aflatoxin exposure assessment may be challenging, as it is dependent on serum levels at specific time points that may not fully capture overall exposure variability. Despite adjusting for various factors, unaccounted confounding may still affect the interpretation of results.

## CONCLUSION

Maternal aflatoxin exposure during pregnancy, despite the presence of maternal EED, was found to be associated with low birth weight and underweight (WAZ score) at birth. Unlike in other longitudinal studies, there was no significant correlation between infant aflatoxin levels and linear growth (length, LAZ) or weight-for-length Z-scores from 3 to 6 months of age, whether or not analyses were adjusted for possible confounders. The age of infants, breastfeeding behaviors, and low exposure to aflatoxin-contaminated foods in early life may account for lower levels of exposure. This finding further supports the importance of exclusive breastfeeding for several months after birth and highlights the importance of promoting national policy actions to minimize aflatoxin contamination of the local food supply, both on farms and in markets.
